# Comorbidity of Attention Deficit Hyperactivity Disorder (ADHD) and Chung‐Jansen Syndrome: Case Report and Review of Literature

**DOI:** 10.1002/ccr3.71593

**Published:** 2025-12-10

**Authors:** Maedeh Khalili, Maryam Yousefi Fahadani, Reza Bidaki, Zahra Talayi

**Affiliations:** ^1^ Department of Biology, Faculty of Science Arak University Arak Iran; ^2^ Student Research Committee Shahid Sadoughi University of Medical Sciences Yazd Iran; ^3^ Research Development Center, Department of Psychiatry, School of Medicine, Shahid Sadoughi Hospital Shahid Sadoughi University of Medical Sciences Yazd Iran; ^4^ Research Center of Addiction and Behavioral Sciences, Department of Psychiatry, Non‐Communicable Diseases Research Institute Shahid Sadoughi University of Medical Sciences Yazd Iran

**Keywords:** attention deficit hyperactivity disorder (ADHD), Chung‐Jansen syndrome, CLCN4 gene, genetic comorbidity, neurodevelopmental disorders, PHIP gene

## Abstract

Chung‐Jansen Syndrome (CJS) is a rare autosomal dominant neurodevelopmental disorder caused by heterozygous mutations in the PHIP gene (6q14.1). It is characterized by developmental delay, intellectual disability, behavioral disturbances including autism spectrum disorder and attention deficit hyperactivity disorder, obesity, and distinct facial dysmorphism such as synophrys and an upturned nose. Although clinical presentations vary, behavioral problems and delayed motor and speech milestones are common. We report a 7‐year‐old male with delayed motor and speech development, synophrys, and an upturned nose—features that are rarely documented in CJS. The patient exhibited significant resistance to multiple psychotropic treatments and presented with severe psychological and neurological symptoms. Whole Exome Sequencing revealed pathogenic mutations in both the PHIP and CLCN4 genes. The PHIP mutation was associated with core neuropsychiatric symptoms, including anxiety and depression, whereas the CLCN4 mutation contributed to severe brain atrophy observed in neuroimaging, further exacerbating cognitive and behavioral deficits. Additional copy number variations (CNVs) were detected, indicating a complex genetic background. The interplay between PHIP and CLCN4 mutations appeared to disrupt neuronal growth and synaptic function, thereby intensifying the clinical phenotype. The constellation of symptoms, including resistance to standard therapies, highlights how genetic overlap can aggravate neurodevelopmental and psychiatric manifestations in CJS. This case underscores the necessity of comprehensive genetic testing, including whole exome sequencing and CNV analysis, in patients with atypical or severe presentations of CJS. The findings emphasize that genetic interactions can amplify clinical severity and advocate for further research into the molecular mechanisms underlying such complex syndromes to improve diagnostic precision and inform targeted therapeutic strategies.

## Key Clinical Message

Clinicians should consider dual genetic diagnoses in rare syndromes presenting with complex psychiatric features such as ADHD, as overlapping mutations can intensify neurodevelopmental symptoms and necessitate early, multidisciplinary evaluation and management.

## 1 Introduction

Chung–Jansen Syndrome (CJS) is a rare genetic disorder first described in 2016–2018 by Chung, Jansen, and colleagues. It is characterized by developmental delay (DD), learning difficulties/intellectual disability (ID), behavioral abnormalities, facial dysmorphism, and obesity. Other reported features include hypotonia and cognitive impairment [1]. CJS is an autosomal dominant disorder caused by haploinsufficiency of PHIP (pleckstrin homology domain interacting protein) [2].

Heterozygous mutations in PHIP are linked to behavioral issues, intellectual disability or developmental delays, obesity or excess weight, and distinct facial abnormalities. In the present case, genetic testing also revealed a mutation in the CLCN4 gene. Dysfunction of CLCN4 can lead to X‐linked intellectual disabilities, ranging from borderline to profound, as well as epilepsy.

Behavioral and psychiatric disorders such as autistic features, mood disorders, obsessive‐compulsive behaviors, and aggression are common in affected children and adults. Psychological manifestations are rare in CJS; here, we report that the coexistence of PHIP and CLCN4 mutations led to severe psychological symptoms [3].

## 2 Case History/Examination

A 7‐year‐old boy, the third child of consanguineous parents, was referred with neurodevelopmental delay, aggressive behavior, weight gain, facial deformities, seizures, intellectual disability, hyperactivity, clinodactyly, semi‐syndactyly, microcephaly, and hypotonia. He was born via vaginal delivery at a birth weight of 2.78 kg.

At 1.5 years, parents noticed progressive weight gain and delayed growth. He began walking at 2.5 years with occupational therapy support. Speech development started normally but was limited to single words by age 4; by age 6, he could speak fluently. The patient also displayed fear and anxiety during daily tasks. Ongoing learning difficulties and behavioral challenges persisted. Discontinuation of medications led to urinary incontinence and sleep difficulties. Prior treatments included citicoline, sodium valproate (6 months–5 years), piracetam, and aripiprazole, which were ineffective.

## 3 Methods (Differential Diagnosis, Investigations, and Treatment)

To identify genetic mutations associated with Chung‐Jansen Syndrome (CJS), Whole Exome Sequencing (WES) was performed using the Illumina platform and Agilent Sure Select V7 library preparation kit at 150× sequencing depth. Raw data were mapped to the GRCh38 reference genome using the Burrows‐Wheeler Aligner (BWA). Variant calling was conducted with the Genome Analysis Toolkit (GATK) to identify Single Nucleotide Polymorphisms (SNPs) and Insertions/Deletions (INDELs). Variants were analyzed using the Palin Var pipeline and classified according to the latest ACMG (American College of Medical Genetics and Genomics) guidelines.

At this stage, non‐neutral variants (which may have clinical effects) were screened and reviewed by the specialized team. In addition to identifying single nucleotide variants (SNVs), the WES data were analyzed for Copy Number Variations (CNVs) to detect larger structural changes in the genome, such as deletions or duplications of chromosomal segments. All genetic data and neuroimaging findings were integrated to assess mutations and structural brain changes. Sequencing data were processed using bioinformatics software to identify and analyze variants and CNVs.

Whole Exome Sequencing (WES) identified mutations in the PHIP gene, consistent with clinical symptoms of CJS, including psychological issues, developmental delay, and obesity. A mutation in the CLCN4 gene was also detected, which contributed to significant brain atrophy on MRI. The PHIP mutation was associated with the patient's core psychological symptoms, while the concurrent CLCN4 mutation exacerbated neurological disorders, brain atrophy, and cognitive deficits. These findings highlight the crucial role of genetic interactions in the severity and presentation of clinical symptoms in CJS.

### 3.1 Differential Diagnosis

The differential diagnoses considered included mental retardation, intellectual disability, autism, celiac disease, and other dysmorphic genetic syndromes. Each was evaluated and ruled out or conditionally confirmed as follows:

#### 3.1.1 Mental Retardation (Intellectual Disability)

- Initial consideration was due to the patient's developmental delays and functional problems.
- Cognitive and functional assessments showed relatively preserved verbal and executive abilities, with a significant gap between verbal and functional skills—not consistent with simple intellectual disability.
- Mental retardation was therefore ruled out as the primary diagnosis, though learning disabilities and developmental delay were considered part of the Chung‐Jansen genetic syndrome.

#### 3.1.2 Intellectual Disability

- Considered due to learning difficulties and delayed motor and verbal skills.
- Further analysis indicated that the patient's issues were primarily due to the specific genetic syndrome, which has distinct behavioral and cognitive features.
- Thus, intellectual disability alone was insufficient as a diagnosis and is considered within the context of CJS.

#### 3.1.3 Autism

- Behavioral problems prompted consideration of autism.
- Detailed behavioral assessment showed absence of key features such as severe social interaction deficits and distinct repetitive behaviors. Dysmorphic features and syndrome‐specific behavioral problems indicated that symptoms were attributable to CJS rather than autism itself.

#### 3.1.4 Celiac Disease

- Considered due to some gastrointestinal or nutritional issues in the patient's history.
- Serological tests and clinical evaluations showed that the patient's gastrointestinal symptoms were not consistent with celiac disease, and all relevant tests were negative.
- Therefore, celiac disease was ruled out as the cause of the patient's symptoms, and nutritional issues were interpreted as secondary to the genetic syndrome and its associated disorders.

#### 3.1.5 Genetic Syndromes and Dysmorphism (Especially Chung‐Jansen Syndrome)

- Given the patient's physical features—including unibrow (synophrys), epicanthal folds, upturned nose, characteristic fingers, and skin spots—along with behavioral problems and developmental delays, CJS was considered the primary diagnosis.
- The diagnosis was confirmed by molecular genetic testing, which identified a mutation in the PHIP gene at 6q14.1.

Other genetic syndromes with similar features—such as Fragile X syndrome, Down syndrome, and Noonan syndrome—were ruled out based on the absence of their characteristic features and results of genetic and karyotype testing (Table 1).

**TABLE 1:** Differential diagnosis of Chung‐Jansen syndrome.

Differential diagnosis	Ruling out or confirmation method	Final result
Mental retardation	Cognitive assessment, skill gap analysis	Ruled out as independent diagnosis
Intellectual disability	Cognitive assessment, syndrome correlation	Part of Chung‐Jansen syndrome
Autism	Specialized behavioral assessment	Ruled out due to incomplete match
Celiac disease	Serological and clinical tests	Ruled out due to negative tests
Genetic syndromes	Molecular genetic tests and karyotype	Chung‐Jansen syndrome confirmed

## 4 Conclusion and Results (Outcome and Follow‐Up)

This report demonstrates that mutations in PHIP and CLCN4 can increase the severity of psychological and neurological symptoms in patients through independent or shared pathways. These findings highlight the importance of considering genetic overlap in the diagnosis, management, and research of rare diseases. Analysis also revealed the presence of CNVs in specific regions of the patient's genome, which may further influence clinical manifestations.

## 5 Discussion

CJS is a rare disorder with complex symptoms, including psychological issues, developmental delay, and obesity [4]. This study reports a patient carrying a PHIP gene mutation (autosomal dominant) as well as an X‐linked CLCN4 gene mutation. The genetic overlap likely contributes to the severity of the patient's psychological and neurological symptoms [5].

Our findings indicate that the PHIP mutation is primarily responsible for core psychological and behavioral symptoms, such as anxiety and depression [6], while the CLCN4 mutation, by causing brain atrophy, exacerbated the severity of the disorder [4]. Psychological and psychiatric symptoms—including severe anxiety, depression, and other neuropsychological issues—are key features of CJS, typically attributed to PHIP mutations, which regulate neuronal and synaptic growth [7].

Consistent with previously reported cases, our patient exhibited severe psychological symptoms, emphasizing the importance of addressing psychological dimensions in managing such genetic disorders [5]. The PHIP mutation contributed primarily to anxiety and depression, with independent effects on brain structure and cognitive function [6].

A notable aspect of this case is the CLCN4 mutation, which significantly affected brain structure, particularly causing brain atrophy, as seen in MRI images [8]. This structural change may further influence the severity of neuropsychiatric symptoms in CJS. The interaction between PHIP and CLCN4 mutations illustrates how genetic overlap can intensify clinical manifestations, highlighting the need for comprehensive genetic assessment in patients with atypical or severe presentations [7].

These findings underscore the importance of integrating genetic, neuroimaging, and clinical data to inform precise diagnosis and targeted management strategies. Recognition of such complex genetic interactions can improve understanding of disease mechanisms and guide individualized therapeutic interventions [5].

The patient's psychological and neurological disturbances are likely influenced by the overlap of PHIP and CLCN4 mutations. The combination of these mutations appears to contribute to the intensification and complexity of psychological and cognitive symptoms [7]. This suggests a combined, potentially additive effect of the mutations, which has not been reported in previous cases [8]. Mutations in these two genes may act through shared or related molecular pathways, including neuronal growth, synaptic transmission, and regulation of neuronal function [6]. While PHIP regulates neuronal and synaptic growth, CLCN4 affects ion channel function in nerve cells, thereby influencing neural network activity [4]. The combination of mutations may disrupt these pathways, leading to severe psychological symptoms [5]. Gender also plays a role, as CLCN4 is located on the X chromosome. In males, who have a single X chromosome, the mutation typically results in more severe effects, whereas in females, random X‐inactivation may modulate the mutation's impact [8]. In our male patient, these effects are more pronounced [7]. This study also highlights the limitations of Whole Exome Sequencing (WES) in detecting larger structural variations, such as CNVs [6]. While WES effectively identifies small variants, larger structural changes require confirmation with methods such as array Comparative Genomic Hybridization (array CGH) or Multiplex Ligation‐dependent Probe Amplification (MLPA) [7]. In this case, CNV data were analyzed, but further investigations are needed for confirmation [8]. These findings emphasize the importance of comprehensive and multifaceted genetic testing in patients with rare disorders [4]. Such approaches help identify complex genetic causes and guide precise treatment strategies [5]. Accurate detection of mutations and their correlation with clinical symptoms can improve patient management and therapeutic outcomes [6] (Table 2).

**TABLE 2:** The review of literature.

Article	Number 1 (7)	Number 2 (8)	Number 3 (6)
Journal	*Molecular Case Studies*	*American Journal of Medical Genetics*	*Human Genetics*
Year	2024	2022	2022
Patient characteristics	28 infected people 15 women and 12 men with the age of 2 to 47 years 13 of them were reported to be infected with Chong Jansen	First patient: A 4.8‐year‐old girl with normal weight and height, but with various mental and physical problems Second patient: 4‐year‐old girl with delayed visual development and visual and mental problems	First patient: 4‐year‐old with developmental disorders and multiple genetic problems Second patient: The mother of the first patient
Family history	No family history was observed among the subjects	No family history was observed among the subjects	Family history was observed among the subjects
Clinical signs	Delayed growth Cognitive disorders Behavioral problems Obesity and eye problems and hypotonia	Delayed growth, mental disability, obesity, changes in appearance, psychological problems, vision problems in both First patient: With delay in motor skills, orthopedic problems, digestive problems, behavioral disorders, mood swings, sensory processing disorder and autism, syndactylic fatigue of the toes Second patient: With chronic constipation	First patient: With facial problems, kidney abnormalities, brain disorders such as thinning of the corpus callosum and delay in the development of movement Second patient: With type 1 diabetes, thyroid disorders, myopathy and growth disorder and low IQ, appearance similar to CDLS
Mutations and genes involved	Study on DNA samples of people with different types of PHIP, gene changes in nonsense, splice, frameshift, missense, such as There was similar methylation overlap in these individuals High degree of overlap observed in clinical features and DNAm patterns of CHUJANS, BFLS, and WHIKERS samples patterns of CHUJANS, BFLS, and WHIKERS samples	Mutation in PHIP gene on chromosome 6Q14.1 DCAF14 protein, a complete isoform of PHIP, plays a role in replication and transition from metaphase to anaphase and stabilization of the replication fork	In patients, gene mutations were identified in MYH7 (which is responsible for hypertrophic cardiomyopathy) and PHIP (which is associated with CHUJANS syndrome) In the first patient, a deletion was identified in the 20p12.1 region that included the FLRT3 and MACROD2 genes, which have been linked to genetic abnormalities such as neurodevelopmental disorders
Diagnostic methods	Examination of DNA methylation abnormality People by way of: Infinum methylation EPIC bead chip microarrays	Investigating the mechanisms involved in stability of replication forks and protection of newly replicated DNA and the role of PHIP protein in replication stress Identification and analysis of two variants P.D488V and P.E963G of PHIP gene in people with Chong‐Johnson syndrome, both of which indicate genome instability	Research and investigation of CDLS disease and its relation Bachong Jansen Using different diagnostic techniques such as Array CGH, WES (Whole Exome Sequencing) and facial recognition software such as Face2Gene Using WES, genetic alterations associated with CHUJANS syndrome and related diseases such as MYH7 and PHIP were identified
Treatment methods	They did not find a specific treatment method	They did not find a specific treatment method	They did not find a specific treatment method

## 6 Conclusion

These results indicate the complexity and genetic diversity in CJS and emphasize the need for detailed investigation of the overlap between mutations in various genes to better understand genetic interactions and develop more effective treatments. In previous studies, comorbidity between this syndrome and significant psychiatric disorders has been rarely reported, making this case one of the first documented instances. Psychiatric issues in this rare genetic disorder have been reported only in limited cases. Our study highlights the comorbidity of the two mentioned genes and their impact on clinical presentation.

## Author Contributions

**Maedeh Khalili:** writing – original draft, writing – review and editing. **Maryam Yousefi Fahadani:** writing – original draft, writing – review and editing. **Reza Bidaki:** investigation, project administration, supervision, validation. **Zahra Talayi:** writing – original draft, writing – review and editing.

## Funding

The authors have nothing to report.

## Ethics Statement

This study was presented to Shahid Sadoughi University of Medical Sciences Committee of Ethics and was confirmed. IR.SSU.MEDICINE.REC.1402.168.

## Consent

The patient provided written informed consent for inclusion in this case report and for the publication of any clinical information that may reveal their identity.

## Data Availability

The original contributions presented in the study are included in the article. Further inquiries can be directed to the corresponding author.

## References

[1] Α. Kampmeier , E. Leitão , I. Parenti , et al., “PHIP‐Associated Chung‐Jansen Syndrome: Report of 23 New Individuals,” Frontiers in Cell and Developmental Biology 10 (2023): 1020609. 36726590 10.3389/fcell.2022.1020609 PMC9886139

[2] G. Albano , K. Rowlands , L. Baciadonna , G. L. Coco , and V. Cardi , “Interpersonal Difficulties in Obesity: A Systematic Review and Meta‐Analysis to Inform a Rejection Sensitivity‐Based Model,” Neuroscience and Biobehavioral Reviews 107 (2019): 846–861. 31585134 10.1016/j.neubiorev.2019.09.039

[3] H. Kaur and I. Panigrahi , “Chung–Jansen Syndrome with obesity,” Obesity Research & Clinical Practice 15 (2021): 303–305. 33867250 10.1016/j.orcp.2021.03.015

[4] S. Li , W. Zhang , P. Liang , et al., “Novel Variants in the CLCN4 Gene Associated With Syndromic X‐Linked Intellectual Disability,” Frontiers in Neurology 14 (2023): 1096969. 37789889 10.3389/fneur.2023.1096969 PMC10542403

[5] E. E. Palmer , T. Stuhlmann , S. Weinert , et al., “De Novo and Inherited Mutations in the X‐Linked Gene CLCN4 Are Associated With Syndromic Intellectual Disability and Seizure and Behavior Disorders in Males and Females,” Molecular Psychiatry 23 (2018): 222–230. 27550844 10.1038/mp.2016.135 PMC5794876

[6] N. Vos , S. Haghshenas , L. van der Laan , et al., “The Detection of a Strong Episignature for Chung‐Jansen Syndrome, Partially Overlapping With Börjeson‐Forssman‐Lehmann and White‐Kernohan Syndromes,” Human Genetics 143 (2024): 761–773. 38787418 10.1007/s00439-024-02679-w PMC11186873

[7] N. Tirado‐Hathaway , C. W. K. Class , C. Chung , and H. Dungrawala , “PHIP Variants Associated With Chung‐Jansen Syndrome Disrupt Replication Fork Stability and Genome Integrity,” Molecular Case Studies 8, no. 5 (2022): a006212. 35863899 10.1101/mcs.a006212 PMC9528965

[8] B. Conti , B. Rinaldi , M. Rimoldi , et al., “Chung‐Jansen Syndrome Can Mimic Cornelia de Lange Syndrome: Another Player Among Chromatinopathies?,” American Journal of Medical Genetics Part A 191, no. 6 (2023): 1586–1592. 36843271 10.1002/ajmg.a.63164

